# The curse of the protein ribbon diagram

**DOI:** 10.1371/journal.pbio.3001901

**Published:** 2022-12-12

**Authors:** Philip E. Bourne, Eli J. Draizen, Cameron Mura

**Affiliations:** 1 School of Data Science, University of Virginia, Charlottesville, Virginia, United States of America; 2 Department of Biomedical Engineering, University of Virginia, Charlottesville, Virginia, United States of America

## Abstract

Does the visual reductionism of the protein ribbon diagram facilitate or hinder scientific insight? In this Perspective, Philip Bourne encourages a reevaluation of its value in an era of machine learning and AI.

## Teaser

Does reductionism, in the era of machine learning and now interpretable AI, facilitate or hinder scientific insight? The protein ribbon diagram, as a means of visual reductionism, is a case in point.

Profound advances in protein structure prediction, and our own recent work on exploring protein fold space, both of which use deep learning methods, got us thinking about something one of us (PEB) has taught for a long time—the curse of the ribbon. Do some reductionist models and/or scientific representations, such as the ribbon diagram illustrating protein structure, facilitate research, only to eventually hinder further insight?

*Science* Magazine chose [1] the AI-driven software, AlphaFold-2 (AF2) [2], as the 2021 Breakthrough of the Year, as it effectively solved a long-standing challenge in molecular biology, namely, predicting a 3D structure of a protein from its 1D sequence. While one can argue the nuances—(i) AF2 might not be solving the protein folding problem since we don’t know the exact mechanism by which folding occurs, (ii) it does not determine the exact structure to the level achieved by experiment for every case, and (iii) at the time it relied upon having many homologs available, e.g., to build multiple sequence alignments (so it’s not predicting from 1 sequence alone, though single-sequence structure prediction is a very active area)—it is still a monumental advance that will influence how we think about protein function, protein design and much more. In short, AF2 and its developers at DeepMind deserve the accolade.

The question then becomes why did AI succeed where humans have failed? Again, there are nuances. Humans have not failed exactly. The brilliantly conceived and executed Critical Assessment of Structure Prediction (CASP), held since 1994 [3], has shown the significant progress in structure prediction over the years; but, all efforts fell far short of what was achieved by AF2 and its predecessor, AlphaFold [4], and by other efforts, notably RoseTTAFold [5]. What do these algorithms “see” that a human does not? Part of the answer to that question is not what is being “seen” but rather how much is being seen. Even the savviest structural biologist, with an eidetic memory, cannot simultaneously hold the number of features of proteins in their head, on par with a well-trained neural network. In a sense, this is analogous to the software engineering principle that “given enough eyeballs, all bugs are shallow” [6]: With enough protein sequence (input) and structure (output) data, a deep model can “learn” a solution, mapping input to output. Maybe another part of the answer is that human neural networks inaccurately (or at least sub optimally) conceive of protein structures as singular, rigid structures (like the frozen ribbons we see on a page in an article), rather than as the fluid, physiologically functional entities that they are in reality—and which a deep neural network can “learn” as an implicit (latent) representation?

A protein structure, whether experimental or theoretical, once known, is described by a set of 3D Cartesian coordinates, where each (x, y, z) coordinate represents the position of an atom. A standard human-readable text format, either PDB or mmCIF [7], provides a list of all atoms and other metadata used to represent the protein. Staring at such a list of numerical data is essentially futile. Early in the history of structural biology, according to Jane Richardson [8], it was Dick Dickerson who was the first to make a protein schematic and Irving Geis the first to show successive peptide planes with ribbons tracing the protein backbone. These diagrams are now the stuff of legend, as they should be, and they can be found on the walls of laboratories and homes of structural biologists. Jane herself, with husband David, illustrated the full range of protein structures with a variety of ribbon diagrams in a landmark 1981 article [9]. That tour de force, from which one of us (PEB) learnt about and became fascinated by, cataloged all 75 protein structures available at the time (there are now 196,979; October 28, 2022).

**Fig 1:**
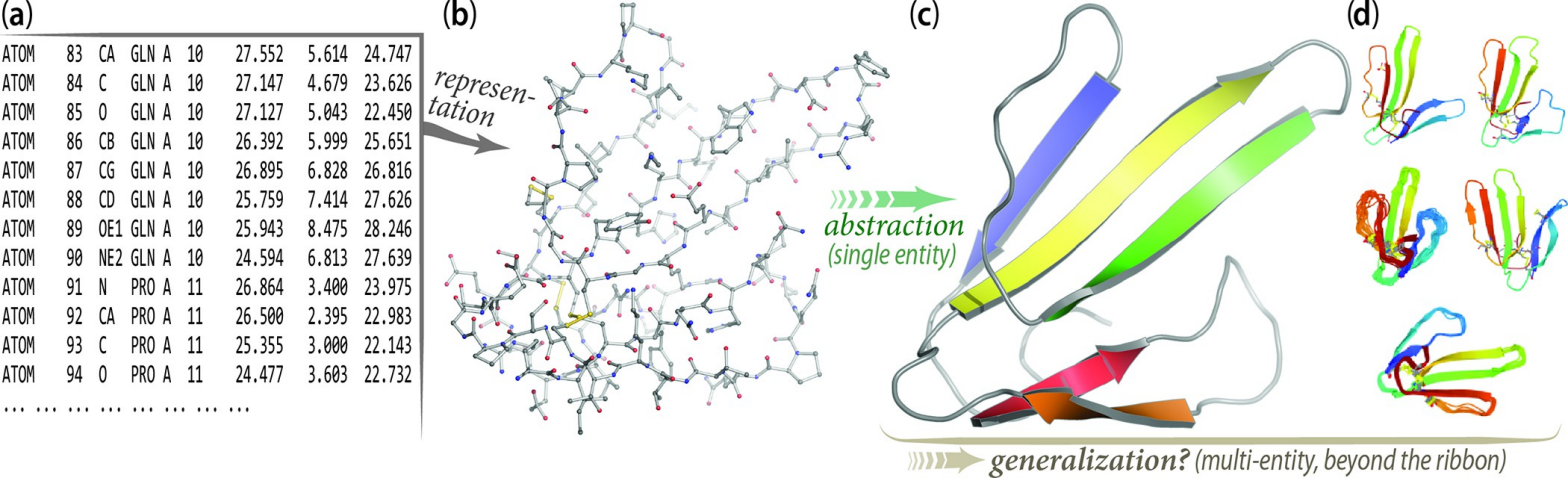
Cartoon ribbon diagrams as a blessing and a curse. The earliest era of structural biology made clear the necessity of molecular visualization for even small proteins, such as the 62-amino acid snake venom toxin shown here (PDB ID 3EBX). In this process, (a) atomic coordinates are visually rendered on a computer display as (b) lines, “sticks,” spheres, etc., thereby creating a representation of the protein’s 3D structure. Though useful for detailed, atomic-scale analyses, e.g., of enzyme mechanisms, such renditions are too visually cluttered and complicated (incomprehensible, essentially) to enable one to grasp a protein’s overall architecture and topology. For that purpose, (c) ribbon diagrams are a blessing: these diagrams are powerful abstractions of a single protein entity, but do they (d) mask other features and relationships.

As is often the case in the biological sciences, comparative analysis proved to be the way forward to understand protein structure. By comparing ribbon diagrams, or similar, initially hand-drawn sketches (and later generated through a variety of increasingly powerful molecular graphics programs), similarities between structures started to become apparent; these 3D spatial “motifs” started to accumulate names like jelly-roll, Greek key, and Rossmann fold as humans drew comparisons to either known objects and patterns, or to the person who first spotted the commonality. As the number of structures increased, the reliance on these simplified visualizations necessarily increased (Fig 1).

With the possible exception of Feynman diagrams, we can’t think of a compact visual representation of scientific information, specific to a given field, that has had more impact on our understanding—in this case, on the relationship between sequence, structure, and function—than the ribbon diagram and variations thereof. In short, it is a blessing. So why are we saying it is a curse, too? We would argue that this singular representational style has become too ingrained in our thinking, to the point non-experts imagine proteins to be really like (static) ribbons. In gazing at ribbon cartoons on a page, we abandon the physicochemical properties that underlie the structure; consider dynamics as only variations of the ribbon; and we think less about solvent, other interacting molecules, cellular location, evolution, and function. In short, the geometric shape, exemplified by the ribbon, dominates our thinking (and, even then, we neglect topography and other geometric features of the surface, e.g., drug-binding pockets and such). There lies the curse. Perhaps it is time to cut the ribbon? Or at least teach an understanding of proteins that has students think beyond the ribbon?

Without ribbon representations of proteins, would humans have solved the protein structure prediction problem? A better question is: has the degree to which we are steeped in thinking about proteins as ribbons limited a type of understanding (models, etc.) of proteins that is necessary to better understand their form and function? There the answer is not so clear. This is exactly why we encourage students to view proteins as collections of bonded atoms undergoing dynamic sets of interactions with each other and the environment—impossible to conceptualize, but the value in opening one’s mind to alternatives would seem important.

Are there other examples where our thinking becomes “locked in”? Taxonomies and ontologies come to mind. The tree of life, while an evolutionary anchor point, is more accurately viewed as dynamic and changeable. If Woese and Fox [10] had not thought so, the discovery of Archaea would have been delayed.

The original anatomy and taxonomy of protein structure [9] was indeed derived partly by a human visual review of ribbon diagrams. This and later classifications were pivotal in our progress in understanding protein sequence-structure-function and evolutionary relationships. Then again, is it in some ways too limiting and restrictive to classify entities such as proteins by placing them into mutually exclusive bins, as is done in existing hierarchical schemes? What if such hierarchical binning has caused us to miss important relationships—for example, relationships arising as shared structural “themes,” which in turn hint at rather distant evolutionary relationships (and suggest deep homology)?

There has been a long debate as to whether the space of all protein folds is discrete or continuous [11]. Current thinking would tend to favor a more continuous model. If that’s the case, the hierarchical binning that occurs in existing classifications might miss important relationships. We posit that indeed we have missed remote linkages, such as between distinct protein “superfolds,” and proposed the existence of the Urfold [12]. An Urfold exists when there is architectural similarity despite topological variability, irrespective of considerations of (known) homology. Ironically, the evidence that suggested the existence of an Urfold was obtained from the visual inspection of many perhaps-related proteins, including ribbon views. More recently, a machine learning study tries to quantitatively “define” the Urfold via learned embeddings in deep generative models, wherein a protein’s sequence, structure, and physicochemical properties can be viewed as being compressed into a lower-dimensional “latent space” representation [13]; though not readily visualized, like ribbon diagrams, such distilled feature representations do suggest a new view of protein relationships. Further scrutiny over time will determine the value of such representations.

What is clear is that machine learning approaches allow us to “look” beyond human digestible metaphors, like the protein ribbon, and will cause us to reevaluate our thinking in many areas of biology. The curse has been lifted in ways we have yet to fully understand.
